# Supplementary figures and images for: Oct4 differentially regulates chromatin opening and enhancer transcription in pluripotent stem cells
Source: eLife. 2022 May 27;11:e71533. doi: 10.7554/eLife.71533 (PMC9142147; doi:10.7554/eLife.71533)

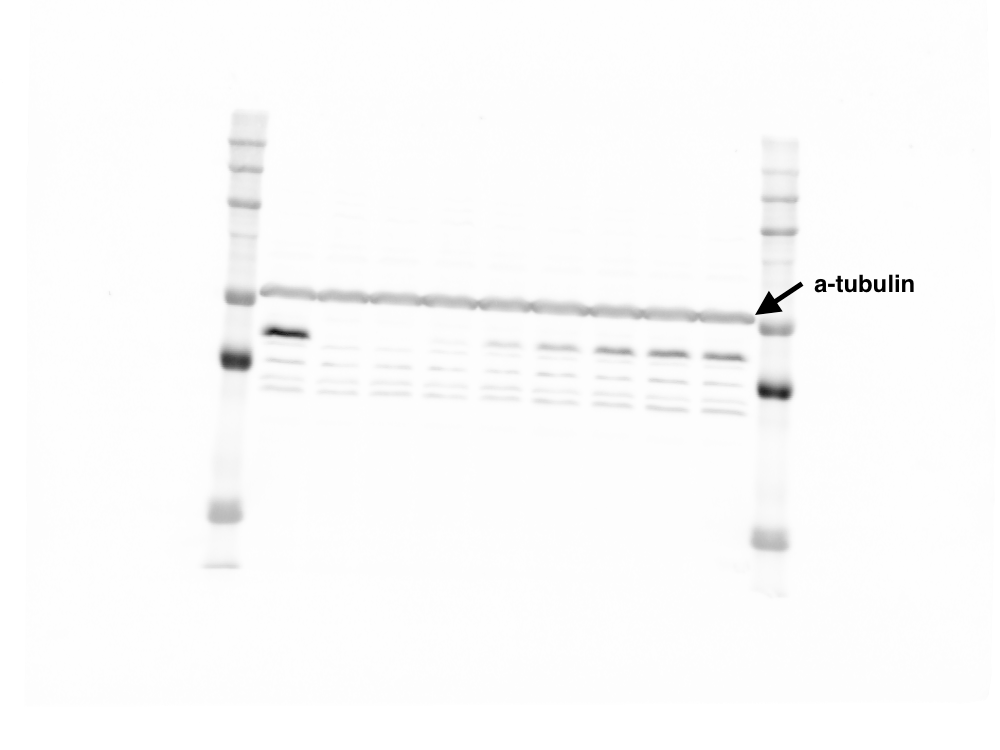

Supplement: Figure 8—source data 1. — The original files of the raw unedited western blots of the whole cell lysate samples and their quantification. [file elife-71533-fig8-data1.zip › Figure 8-source data 1/Figure 8-source data 1-Labelled/2021-10-19 WB Figure 8A a-tubulin marked.tif]

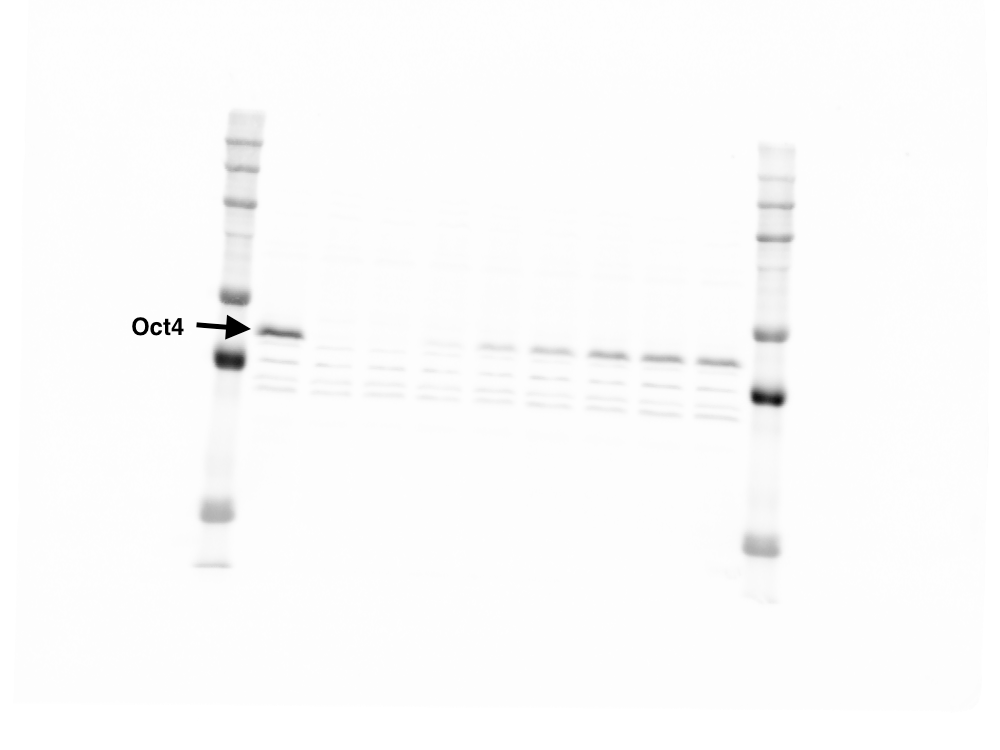

Supplement: Figure 8—source data 1. — The original files of the raw unedited western blots of the whole cell lysate samples and their quantification. [file elife-71533-fig8-data1.zip › Figure 8-source data 1/Figure 8-source data 1-Labelled/2021-10-19 WB Figure 8A Oct4 marked.tif]

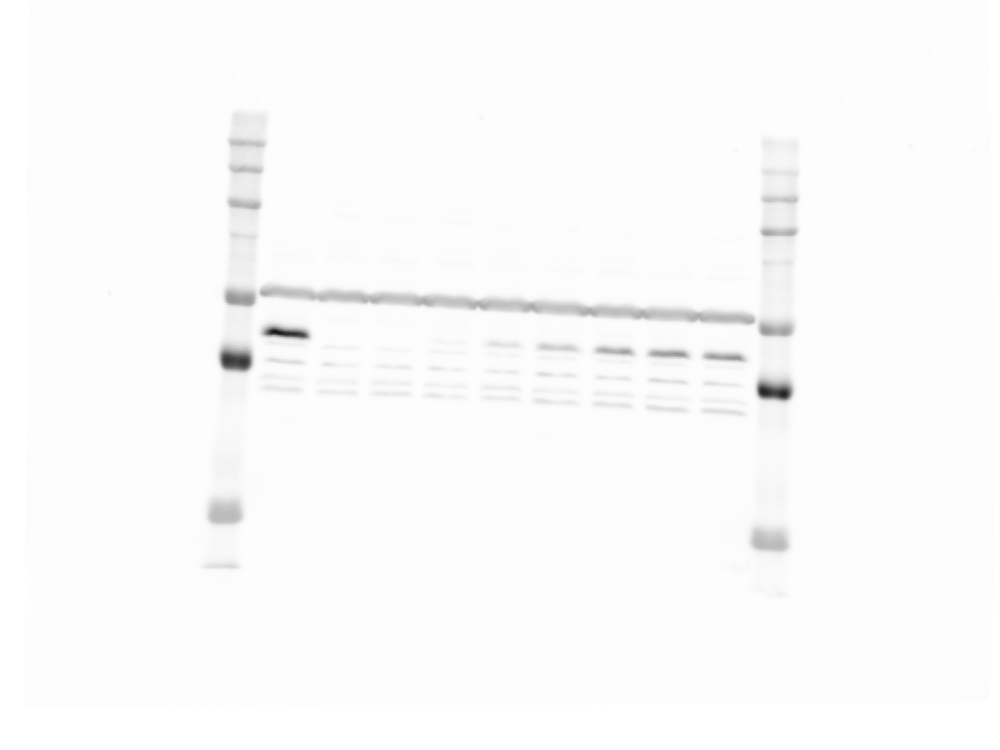

Supplement: Figure 8—source data 1. — The original files of the raw unedited western blots of the whole cell lysate samples and their quantification. [file elife-71533-fig8-data1.zip › Figure 8-source data 1/Figure 8-source data 1-Original/2021-10-19 WB Figure 8A a-tubulin.tif]

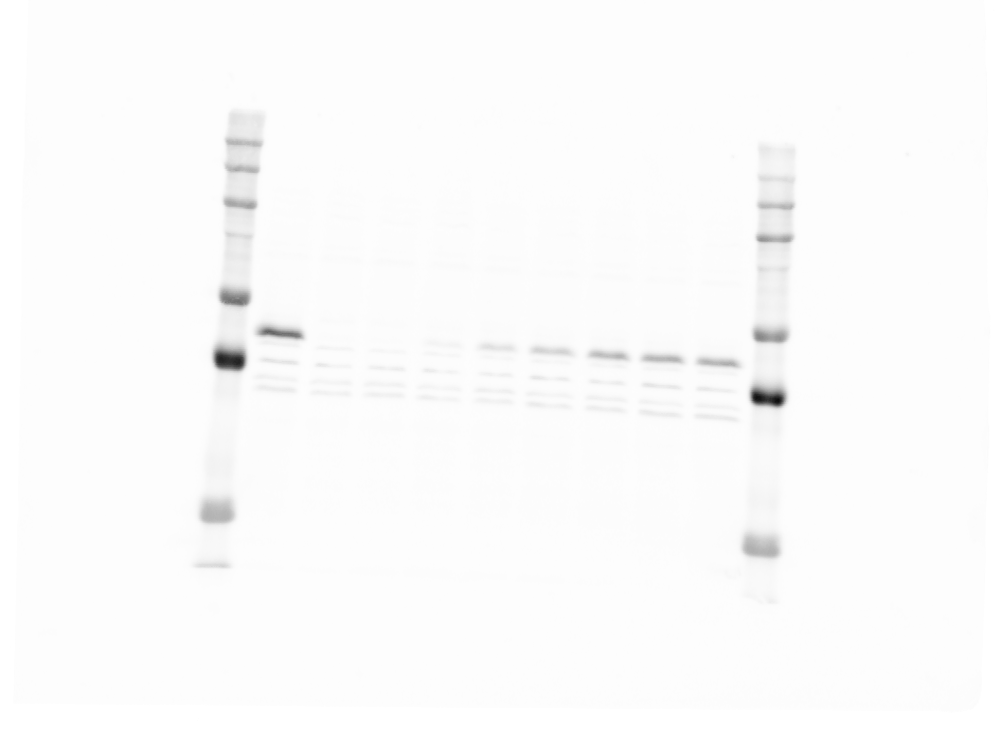

Supplement: Figure 8—source data 1. — The original files of the raw unedited western blots of the whole cell lysate samples and their quantification. [file elife-71533-fig8-data1.zip › Figure 8-source data 1/Figure 8-source data 1-Original/2021-10-19 WB Figure 8A Oct4.tif]

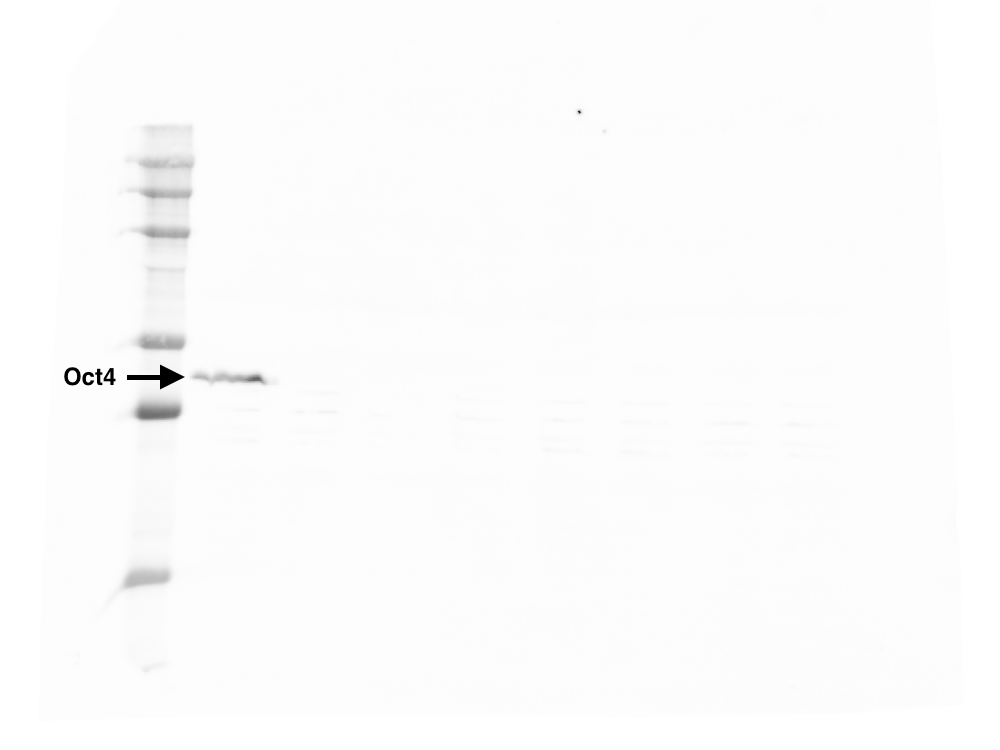

Supplement: Figure 8—figure supplement 1—source data 1. — The original files of the raw unedited western blots. [file elife-71533-fig8-figsupp1-data1.zip › Figure 8-source data 2/Figure 8-figure supplement 1A/Labelled/2021-08-26 WB Figure S8A Oct4 marked.tif]

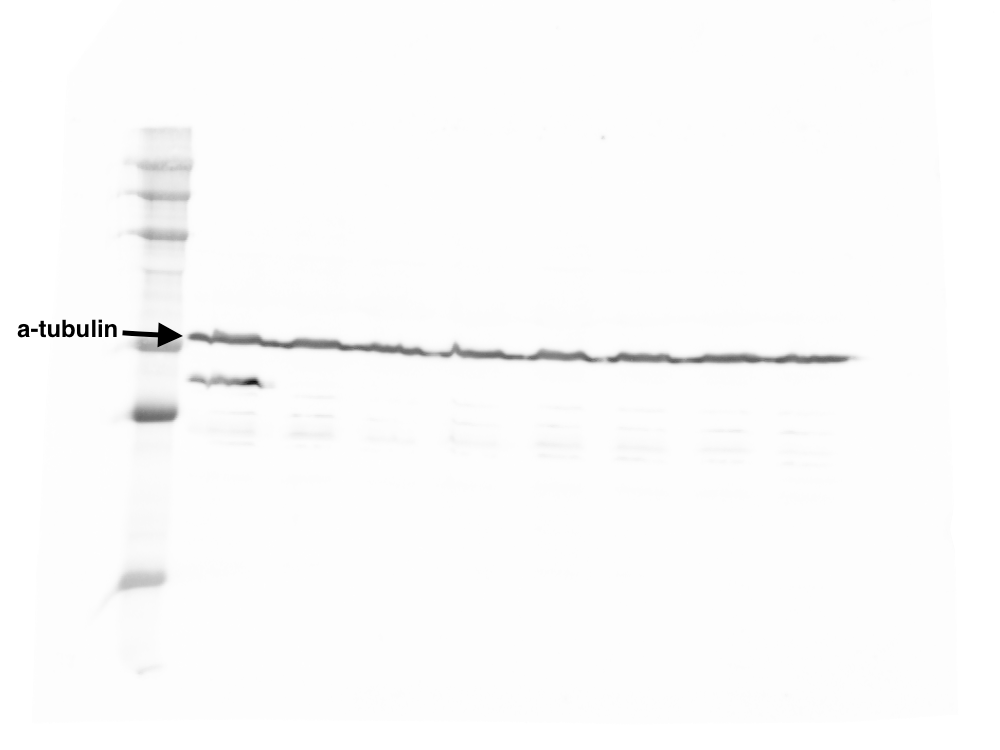

Supplement: Figure 8—figure supplement 1—source data 1. — The original files of the raw unedited western blots. [file elife-71533-fig8-figsupp1-data1.zip › Figure 8-source data 2/Figure 8-figure supplement 1A/Labelled/2021-08-27 WB Figure S8A a-tubulin marked.tif]

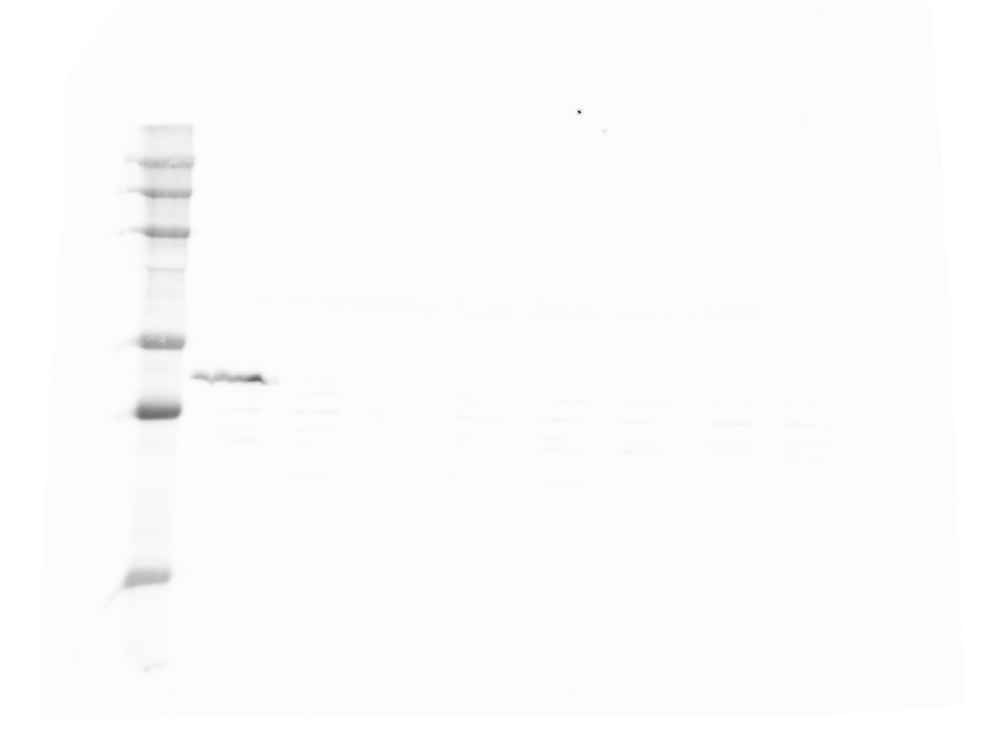

Supplement: Figure 8—figure supplement 1—source data 1. — The original files of the raw unedited western blots. [file elife-71533-fig8-figsupp1-data1.zip › Figure 8-source data 2/Figure 8-figure supplement 1A/Original/2021-08-26 WB Figure S8A Oct4.tif]

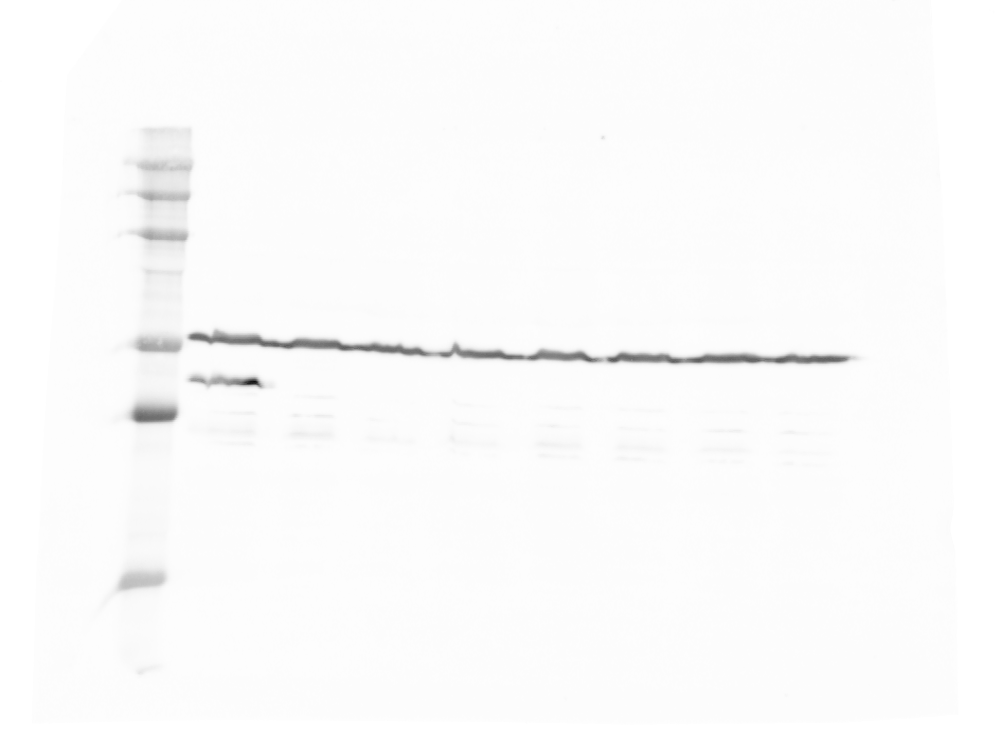

Supplement: Figure 8—figure supplement 1—source data 1. — The original files of the raw unedited western blots. [file elife-71533-fig8-figsupp1-data1.zip › Figure 8-source data 2/Figure 8-figure supplement 1A/Original/2021-08-27 WB Figure S8A a-tubulin.tif]

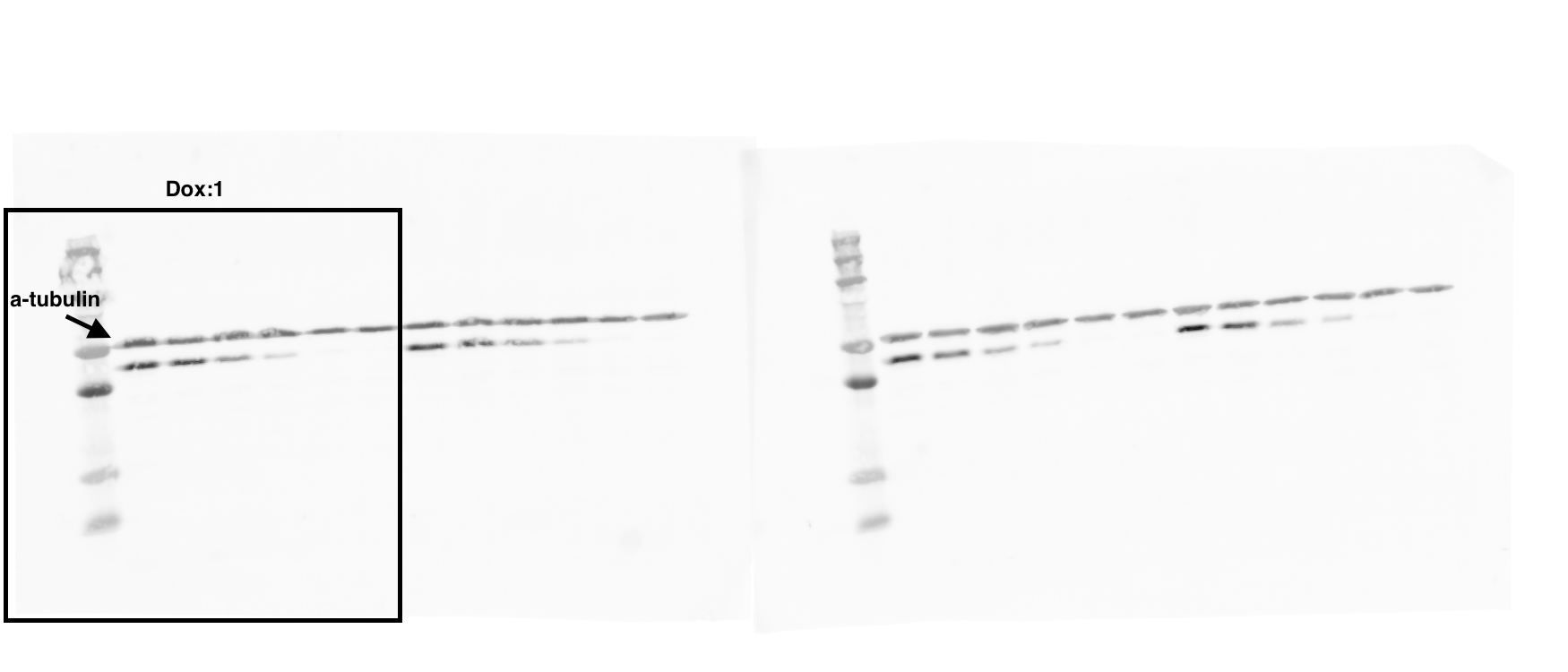

Supplement: Figure 8—figure supplement 1—source data 1. — The original files of the raw unedited western blots. [file elife-71533-fig8-figsupp1-data1.zip › Figure 8-source data 2/Figure 8-figure supplement 1B/Labelled/2021-09-19 WB time course 1_10_100_300 Figure S8B a-tubulin marked.tif]

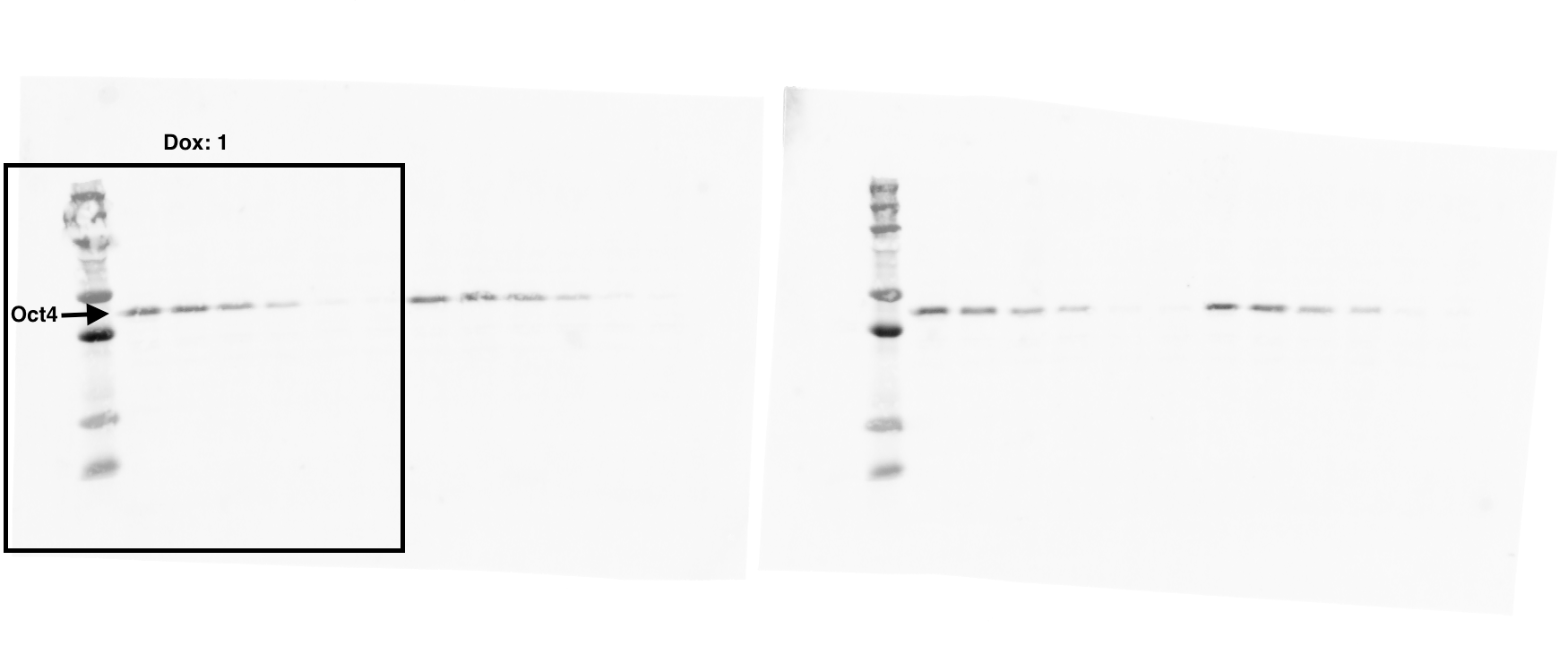

Supplement: Figure 8—figure supplement 1—source data 1. — The original files of the raw unedited western blots. [file elife-71533-fig8-figsupp1-data1.zip › Figure 8-source data 2/Figure 8-figure supplement 1B/Labelled/2021-09-19 WB time course 1_10_100_300 Figure S8B Oct4 marked.tif]

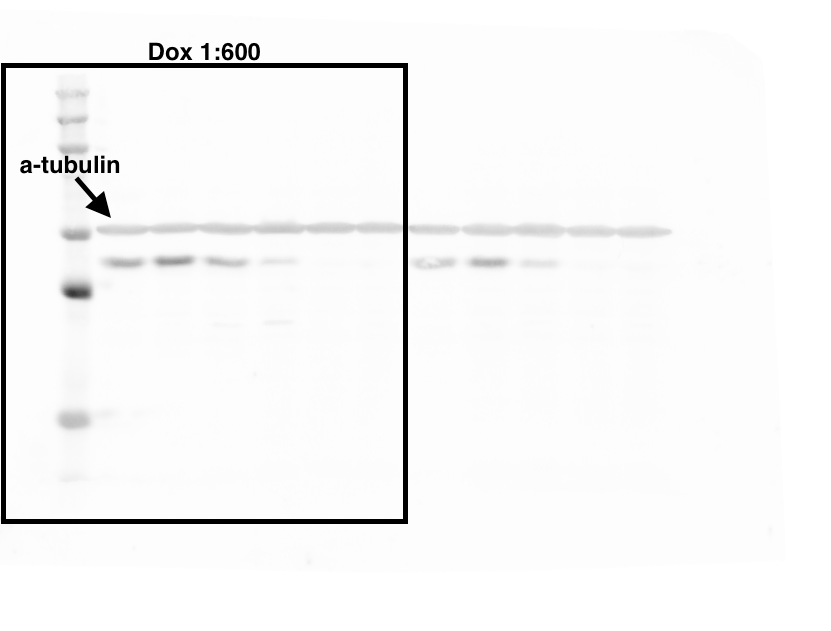

Supplement: Figure 8—figure supplement 1—source data 1. — The original files of the raw unedited western blots. [file elife-71533-fig8-figsupp1-data1.zip › Figure 8-source data 2/Figure 8-figure supplement 1B/Labelled/2021-09-27 WB 600 Figure S8B a-tubulin marked.tif]

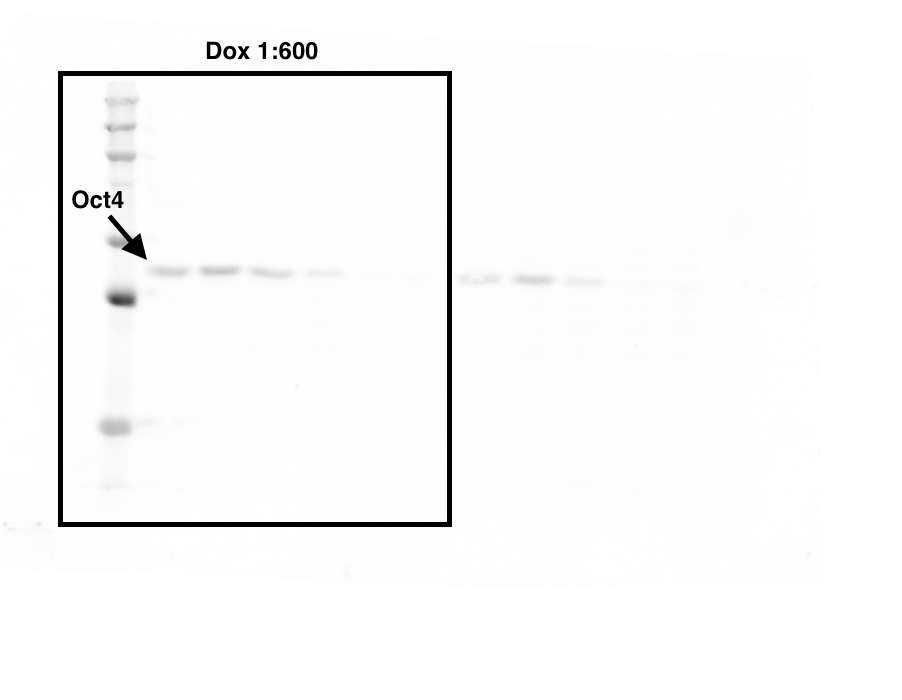

Supplement: Figure 8—figure supplement 1—source data 1. — The original files of the raw unedited western blots. [file elife-71533-fig8-figsupp1-data1.zip › Figure 8-source data 2/Figure 8-figure supplement 1B/Labelled/2021-09-27 WB 600 Figure S8B Oct4 marked.tif]

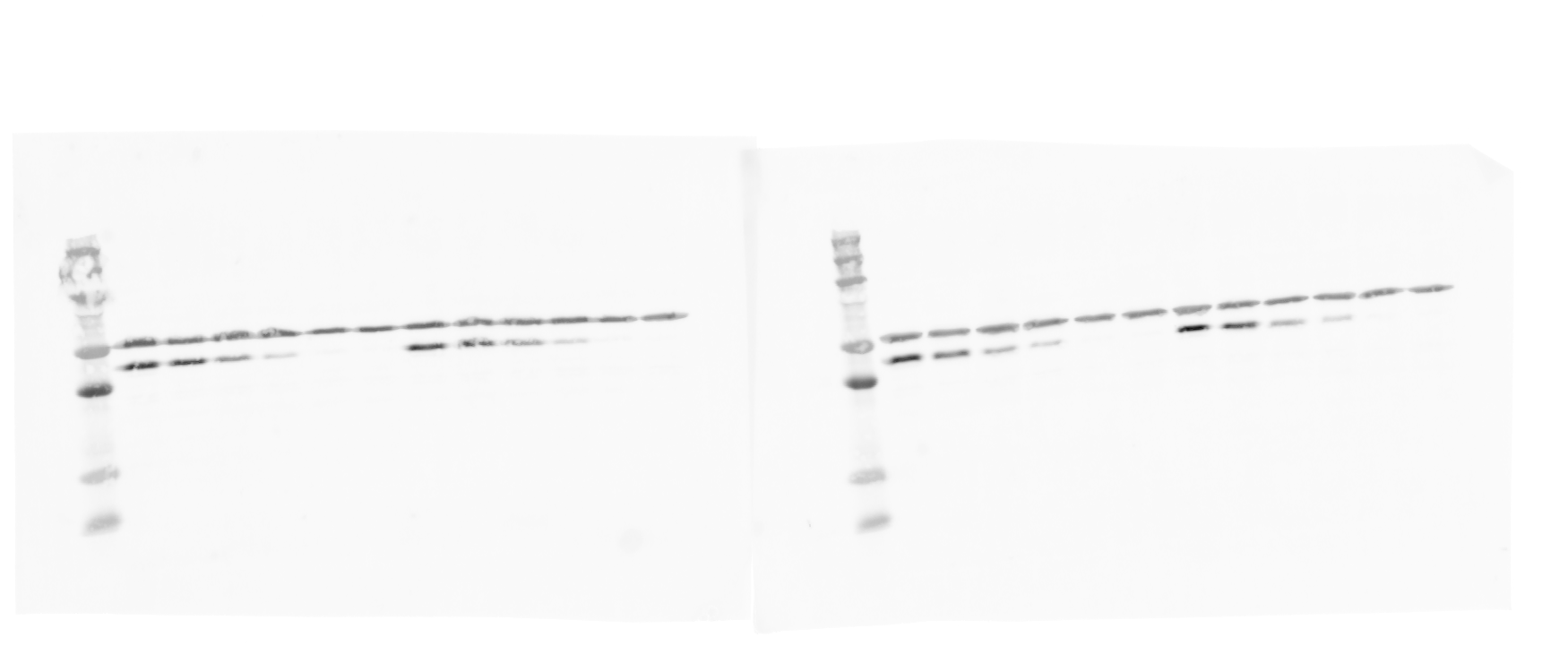

Supplement: Figure 8—figure supplement 1—source data 1. — The original files of the raw unedited western blots. [file elife-71533-fig8-figsupp1-data1.zip › Figure 8-source data 2/Figure 8-figure supplement 1B/Original/2021-09-19 WB time course 1_10_100_300 Figure S8B a-tubulin.tif]

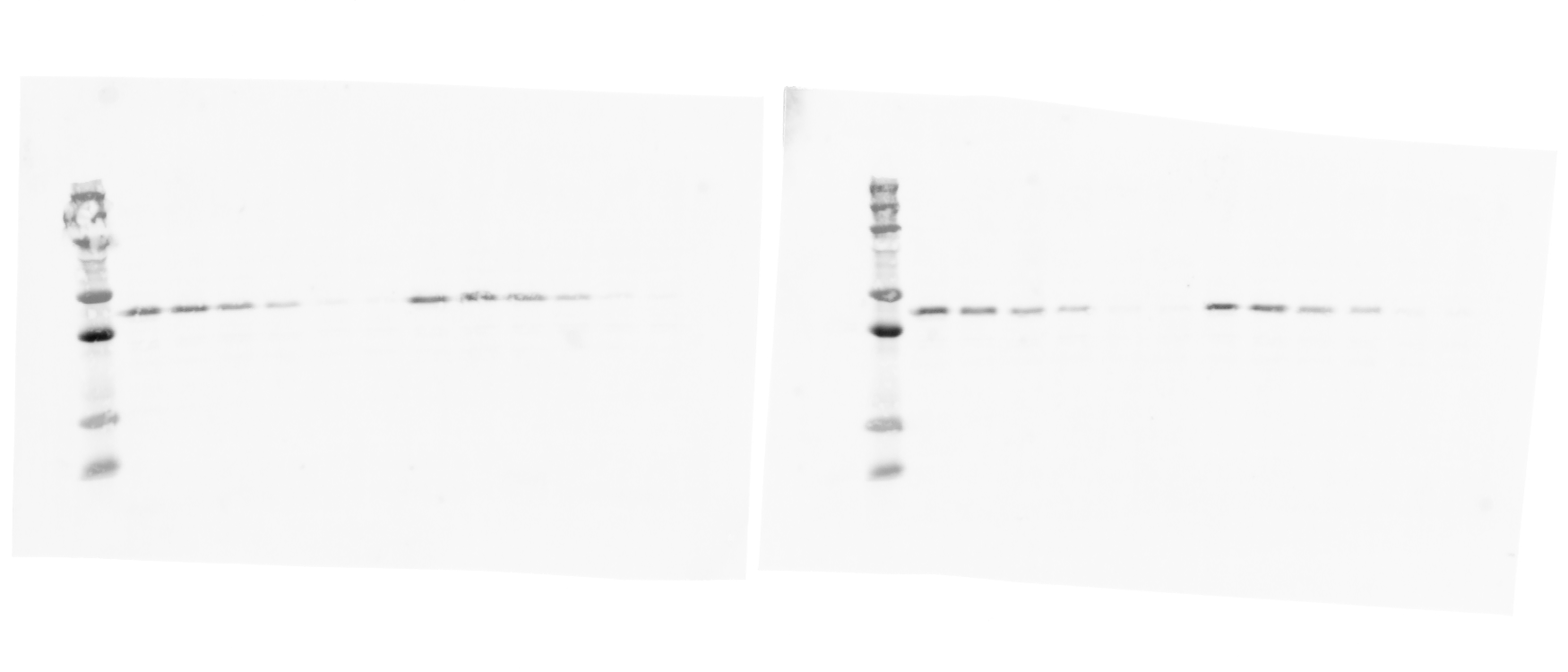

Supplement: Figure 8—figure supplement 1—source data 1. — The original files of the raw unedited western blots. [file elife-71533-fig8-figsupp1-data1.zip › Figure 8-source data 2/Figure 8-figure supplement 1B/Original/2021-09-19 WB time course 1_10_100_300 Figure S8B Oct4.tif]

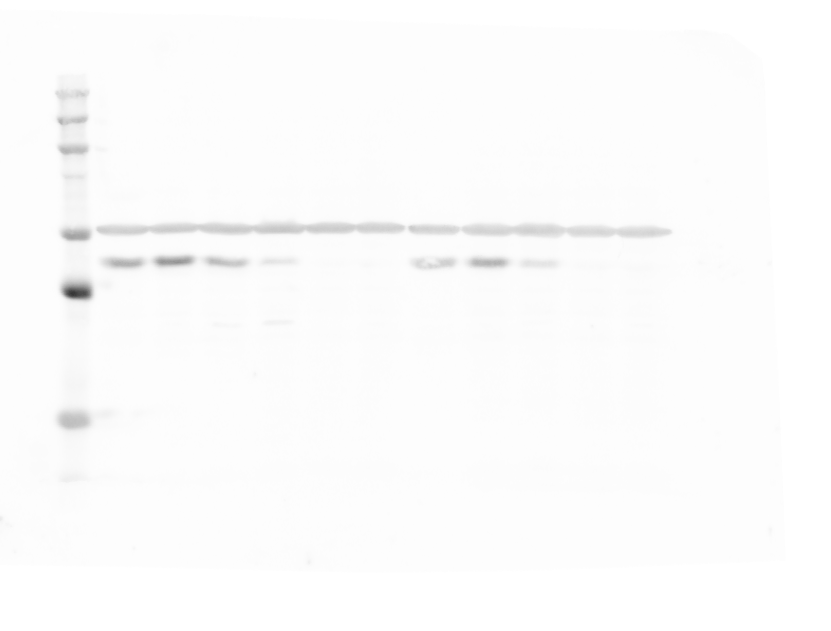

Supplement: Figure 8—figure supplement 1—source data 1. — The original files of the raw unedited western blots. [file elife-71533-fig8-figsupp1-data1.zip › Figure 8-source data 2/Figure 8-figure supplement 1B/Original/2021-09-27 WB 600 Figure S8B a-tubulin.tif]

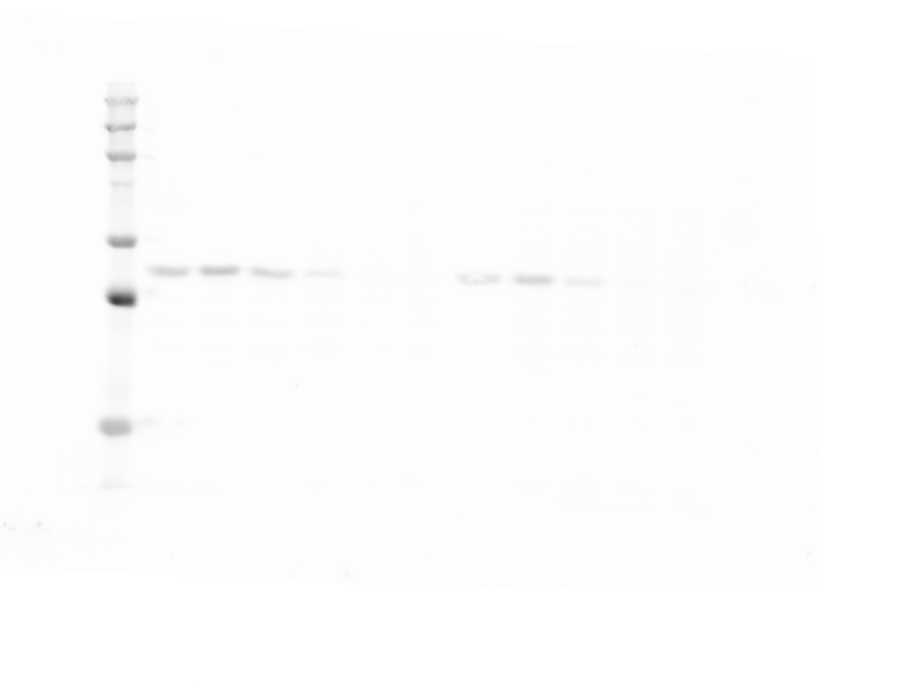

Supplement: Figure 8—figure supplement 1—source data 1. — The original files of the raw unedited western blots. [file elife-71533-fig8-figsupp1-data1.zip › Figure 8-source data 2/Figure 8-figure supplement 1B/Original/2021-09-27 WB 600 Figure S8B Oct4.tif]
