# Supplementary material for: Oct4 differentially regulates chromatin opening and enhancer transcription in pluripotent stem cells
Source: eLife. 2022 May 27;11:e71533. doi: 10.7554/eLife.71533 (PMC9142147; doi:10.7554/eLife.71533)
Supplement: Supplementary file 1. [file elife-71533-supp1.docx]

**Supplementary File 1. Sequencing statistics of TT-seq samples generated in this study, related to Figure 1.**

All samples were sequenced on a NEXTseq 550 sequencing platform in 42bp paired-end mode.

| No. | Hours of DOX treatment | Replicate no. | Sequenced  reads | Mapped reads | Duplicates  （%） |
| --- | --- | --- | --- | --- | --- |
| 1 | 0h | 1 | 139,767,320 | 115,716,286 | 42.6 |
| 2 |  | 2 | 137,196,527 | 115,935,348 | 31.8 |
| 3 | 3h | 1 | 134,759,731 | 113,952,490 | 31.3 |
| 4 |  | 2 | 140,542,668 | 119,816,299 | 35.4 |
| 5 | 6h | 1 | 139,202,151 | 117,349,804 | 38.5 |
| 6 |  | 2 | 139,904,081 | 118,716,899 | 33.7 |
| 7 | 9h | 1 | 143,258,532 | 120,721,286 | 42.5 |
| 8 |  | 2 | 131,906,425 | 110,880,848 | 35.2 |
| 9 | 12h | 1 | 136,024,166 | 115,730,201 | 38.3 |
| 10 |  | 2 | 136,854,827 | 116,897,968 | 33.4 |
| 11 | 15h | 1 | 141,129,470 | 120,097,702 | 40.9 |
| 12 |  | 2 | 132,219,606 | 111,909,955 | 31.6 |
