# Supplementary material for: Oct4 differentially regulates chromatin opening and enhancer transcription in pluripotent stem cells
Source: eLife. 2022 May 27;11:e71533. doi: 10.7554/eLife.71533 (PMC9142147; doi:10.7554/eLife.71533)
Supplement: Supplementary file 2. [file elife-71533-supp2.docx]

**Supplementary File 2. Sequencing statistics of ATAC-seq samples generated in this study, related to Figure 1.**

All samples were sequenced on a NEXTseq 550 sequencing platform in 76bp paired-end mode.

| No. | Hours of DOX treatment | Replicate  no. | Sequenced  reads | Mapped reads | Duplicates  （%） |
| --- | --- | --- | --- | --- | --- |
| 1 | 0h | 1 | 75,879,782 | 60,778,817 | 7.0 |
| 2 |  | 2 | 84,285,910 | 66,234,761 | 7.0 |
| 3 | 3h | 1 | 99,108,163 | 76,192,137 | 7.0 |
| 4 |  | 2 | 95,058,225 | 75,287,164 | 8.0 |
| 5 | 6h | 1 | 100,888,477 | 80,437,745 | 7.0 |
| 6 |  | 2 | 96,527,924 | 77,644,843 | 7.0 |
| 7 | 9h | 1 | 98,029,091 | 76,592,320 | 6.0 |
| 8 |  | 2 | 96,395,215 | 76,365,304 | 7.0 |
| 9 | 12h | 1 | 104,560,309 | 82,912,008 | 7.0 |
| 10 |  | 2 | 91,177,600 | 72,588,902 | 7.0 |
| 11 | 15h | 1 | 93,561,692 | 73,702,559 | 8.0 |
| 12 |  | 2 | 82,330,900 | 65,056,516 | 6.0 |
