# Supplementary material for: Oct4 differentially regulates chromatin opening and enhancer transcription in pluripotent stem cells
Source: eLife. 2022 May 27;11:e71533. doi: 10.7554/eLife.71533 (PMC9142147; doi:10.7554/eLife.71533)
Supplement: Supplementary file 4. [file elife-71533-supp4.docx]

**Supplementary File 4.** **List of previously published ChIP-seq datasets used in this study, related to Figure 3.**

| No. | Read mode | Data | Hours of DOX treatment | Cell line | Available at |
| --- | --- | --- | --- | --- | --- |
| 1 | Paired-end | Oct4 | 0h | ZHBTc4 | NCBI Gene Expression Omnibus (GSE87822) |
|  |  |  | 24h |  |  |
| 2 | Paired-end | Sox2 | 0h | ZHBTc4 | NCBI Gene Expression Omnibus (GSE87822) |
|  |  |  | 24h |  |  |
| 3 | Paired-end | Nanog | 0h | ZHBTc4 | NCBI Gene Expression Omnibus (GSE87822) |
|  |  |  | 24h |  |  |
| 4 | Single-end | Klf4 |  | C57BL/6J | NCBI Gene Expression Omnibus (GSE 90895) |
| 5 | Single-end | Esrrb |  | C57BL/6J | NCBI Gene Expression Omnibus (GSE 90895) |
| 6 | Single-end | H3K4me1 |  | C57BL/6J | NCBI Gene Expression Omnibus (GSE 90895) |
| 7 | Single-end | H3K4me3 |  | C57BL/6J | NCBI Gene Expression Omnibus (GSE 90895) |
| 8 | Single-end | H3K27ac |  | C57BL/6J | NCBI Gene Expression Omnibus (GSE 90895) |
