# Supplementary material for: Oct4 differentially regulates chromatin opening and enhancer transcription in pluripotent stem cells
Source: eLife. 2022 May 27;11:e71533. doi: 10.7554/eLife.71533 (PMC9142147; doi:10.7554/eLife.71533)
Supplement: Supplementary file 5. [file elife-71533-supp5.docx]

**Supplementary File 5. Sequencing statistics of Oct4 ChIP-seq samples generated in this study, related to Figure4 and Figure 7**

All samples were sequenced on a NEXTseq 550 sequencing platform in 43bp paired-end mode.

| No. | Hours of DOX treatment | Replicate  no. | Sequenced  reads | Mapped reads | Duplicates  （%） |
| --- | --- | --- | --- | --- | --- |
| 1 | 0h | 1 | 45,489,989 | 37,572,722 | 8.0 |
| 2 |  | 2 | 44,844,975 | 35,827,918 | 9.0 |
| 3 | 3h | 1 | 45,200,637 | 37,381,092 | 7.0 |
| 4 |  | 2 | 40,712,696 | 33,473,643 | 7.0 |
| 5 | 6h | 1 | 35,615,780 | 28,101,180 | 11.0 |
| 6 |  | 2 | 44,105,296 | 34,059,983 | 12.0 |
| 7 | 9h | 1 | 39,889,543 | 32,484,669 | 8.0 |
| 8 |  | 2 | 33,968,970 | 27,705,901 | 8.0 |
| 9 | 12h | 1 | 49,893,843 | 40,471,411 | 9.0 |
| 10 |  | 2 | 40,182,623 | 31,715,336 | 11.0 |
| 11 | 15h | 1 | 54,230,702 | 43,871,081 | 8.0 |
| 12 |  | 2 | 53,405,989 | 38,050,210 | 10.0 |
