# Supplementary material for: Oct4 differentially regulates chromatin opening and enhancer transcription in pluripotent stem cells
Source: eLife. 2022 May 27;11:e71533. doi: 10.7554/eLife.71533 (PMC9142147; doi:10.7554/eLife.71533)
Supplement: Supplementary file 6. [file elife-71533-supp6.docx]

**Supplementary File 6. Sequencing statistics of Sox2 ChIP-seq samples generated in this study, related to Figure 6-7**

All samples were sequenced on a HiSeq 1500 (Illumina) sequencing platform in 76bp paired-end mode.

| No. | Hours of DOX treatment | Replicate  no. | Sequenced  reads | Mapped reads | Duplicates  （%） |
| --- | --- | --- | --- | --- | --- |
| 1 | 0h | 1 | 31,157,808 | 26,708,870 | 8.0 |
| 2 |  | 2 | 30,358,437 | 26,105,044 | 8.0 |
| 3 | 3h | 1 | 29,224,107 | 25,024,914 | 8.0 |
| 4 |  | 2 | 23,143,269 | 19,813,671 | 8.0 |
| 5 | 6h | 1 | 32,774,215 | 28,167,501 | 8.0 |
| 6 |  | 2 | 26,661,227 | 22,987,161 | 7.0 |
| 7 | 9h | 1 | 29,314,133 | 25,170,019 | 9.0 |
| 8 |  | 2 | 25,428,443 | 21,723,313 | 8.0 |
| 9 | 12h | 1 | 26,165,258 | 22,383,270 | 9.0 |
| 10 |  | 2 | 24,258,711 | 20,855,734 | 8.0 |
| 11 | 15h | 1 | 26,728,664 | 22,835,623 | 8.0 |
| 12 |  | 2 | 24,926,639 | 21,369,856 | 9.0 |
