# Supplementary material for: Oct4 differentially regulates chromatin opening and enhancer transcription in pluripotent stem cells
Source: eLife. 2022 May 27;11:e71533. doi: 10.7554/eLife.71533 (PMC9142147; doi:10.7554/eLife.71533)
Supplement: Supplementary file 7. [file elife-71533-supp7.docx]

**Supplementary File 7. Sequencing statistics of TT-seq samples generated in Oct4 recovery experiments, related to Figure 8.**

All samples were sequenced on a NEXTseq 550 sequencing platform in 42bp paired-end mode.

| No. | Hours of DOX treatment or removal (-) | Replicate no. | Sequenced  reads | Mapped reads | Duplicates  （%） |
| --- | --- | --- | --- | --- | --- |
| 1 | 0h | 1 | 162,486,442 | 135,154,688 | 35.2 |
| 2 |  | 2 | 151,500,959 | 128,252,544 | 35.2 |
| 3 | 15h | 1 | 159,355,676 | 136,220,124 | 34.6 |
| 4 |  | 2 | 168,853,644 | 146,153,356 | 30.0 |
| 5 | -4.5h | 1 | 145,699,511 | 123,005,354 | 34.2 |
| 6 |  | 2 | 191,025,358 | 161,033,031 | 33.9 |
| 7 | -9h | 1 | 184,302,636 | 154,722,822 | 42.5 |
| 8 |  | 2 | 188,316,374 | 161,925,966 | 28.4 |
