# Supplementary material for: Oct4 differentially regulates chromatin opening and enhancer transcription in pluripotent stem cells
Source: eLife. 2022 May 27;11:e71533. doi: 10.7554/eLife.71533 (PMC9142147; doi:10.7554/eLife.71533)
Supplement: Supplementary file 8. [file elife-71533-supp8.docx]

**Supplementary File 8. Sequencing statistics of ATAC-seq samples generated in Oct4 recovery experiments, related to Figure 8.**

All samples were sequenced on a NEXTseq 550 sequencing platform in 76bp paired-end mode.

| No. | Hours of DOX treatment or removal (-) | Replicate no. | Sequenced  reads | Mapped reads | Duplicates  （%） |
| --- | --- | --- | --- | --- | --- |
| 1 | 0h | 1 | 61,304,701 | 48,517,371 | 11.0 |
| 2 |  | 2 | 49,717,771 | 39,893,232 | 10.0 |
| 3 | 15h | 1 | 45,573,913 | 36,530,407 | 12.0 |
| 4 |  | 2 | 63,105,771 | 50,561,954 | 13.0 |
| 5 | -4.5h | 1 | 62,008,093 | 49,911,855 | 12.0 |
| 6 |  | 2 | 52,059,111 | 42,132,656 | 10.0 |
| 7 | -9h | 1 | 57,855,487 | 46,475,503 | 14.0 |
| 8 |  | 2 | 81,920,420 | 66,153,616 | 14.0 |
